# False measurement of glycated hemoglobin in patients without hemoglobin A

**DOI:** 10.1042/BSR20180128

**Published:** 2019-01-30

**Authors:** Minghuan Suo, Dongmei Wen, Weijia Wang, Decai Zhang, Shengnan Xu, Xia Wang, Ting Hu

**Affiliations:** Division of Clinical Laboratory, Zhongshan Hospital of Sun Yat-sen University, Zhongshan, Guangdong 528403, China

**Keywords:** capillary electrophoresis, diabetes mellitus, False measurement, glycated hemoglobin, HPLC

## Abstract

**Background**: Hemoglobin (Hb) A_1c_, a biochemical marker widely used in monitoring diabetes mellitus, can be quantitatively measured by various examining systems. However, significant errors still exist. In the present study, we evaluated the HbA_1c_ level in five patients with compound heterozygotes by five different examining systems and our goal is to identify the existence of erroneous HbA_1c_ measurement.

**Methods:** Blood samples collected from normal (no hemoglobin variants) and abnormal (compound heterozygotes) patients were analyzed by capillary electrophoresis technique and sequence analysis. The samples without HbA expression via above methods were further analyzed for HbA_1c_ by ion exchange HPLC Variant II/ Variant II Turbo 2.0 (VII and VII-T 2.0), boronate affinity HPLC, capillary electrophoresis, and Tinaquant immunoassay.

**Results:** HbA_1c_ expression were unexpectedly detected in the compound heterozygous samples by using additional examining systems: The HPLC VII and VII-T 2.0 detected HbA_1c_ expression in two of five samples and failed to detect the abnormal HbA_2_ expression; the CE system detected HbA_1c_ expression in one of five samples with abnormal HbA_2_ expression; the Ultra2 and PPI system detected the HbA_1c_ expression of all samples without abnormal HbA_2_.

**Conclusions:** Five human samples without HbA expression were additionally detected with HbA_1c_ expression with or without abnormal HbA_2_ expression by five analysis systems and the different examining assay potentially affected the test results. These results demonstrated that the limitations of current examining systems for monitoring patients with hemoglobin disorders highlighting the further improvement in the method of clinical HbA examination.

## Introduction

Glycated hemoglobin (HbA_1c_), the glycated fraction of hemoglobin A, is a biochemical marker. The protein is formed via nonenzymatic glycation of the valine residue at the N-terminal of hemoglobin β-chain with glucose. HbA_1c_ test is routinely utilized in monitoring long-term glycemic control and assessing the risk of complications [1,2]. In the 2010 guideline of the American Diabetes Association, HbA_1c_ was recommended as one criterion for diabetes screening and diagnosis using a cut-off value of 6.5% (48 mmol/mol) [3]. Therapeutic strategies, according to HbA_1c_ test, have been established as well [4]. Therefore, the accurate and precise measurement of HbA_1c_ is extremely crucial to clinical practices. Currently, a variety of methods based on different principles are used for HbA_1c_ measurement in clinical laboratories and these methodologies included cation exchange-high performance liquid chromatography (CE-HPLC), boronate affinity high-performance liquid chromatography (BAC), capillary electrophoresis (CE), and immunoassay. However, the results of HbA_1c_ test from these methods may be influenced with patients’ pathophysiological conditions, such as hemolysis, reduced erythrocyte life span, technical interference of certain hemoglobin variants, or elevated HbF expression [5]. More recently, several studies, reported the measurement of HbA_1c_, were significantly affected by patients with Hb variants (HbAS, HbAE, HbAC, HbAD, HbAJ (Bangkok), HbAG (Taibei)) and increasing evidence suggested that the results of HbA_1c_ in Hb variants detected by implementation of BAC method as well as Roche Tinaquant immunoassay were much less affected than the result of International Federation of Clinical Chemistry and Laboratory Medicine (IFCC) reference method [6–9].

Compound heterozygosity is one of the critical elements to cause genetic disease in human being. The measurement of HbA_1c_ is closely associated with the screen or diagnosis of compound heterozygosity patients with Hb variants. Notably, few studies were reported in the HbA_1c_ measurement from compound heterozygous patients without HbA expression by using these examining assays. In the present study, HbA_1c_ in the blood samples collected from five compound heterozygous patients without HbA expression was analyzed by five common HbA_1c_ detection systems to better understand the HbA_1c_ measurement in clinical application.

## Materials and methods

### Samples

The study was approved by the Ethics Committee of Zhongshan Hospital of Sun Yat-sen University. A total of 40 whole blood samples with EDTA collected from previously tested specimens were conducted with HbA_1c_ analysis by a Bio–Rad Variant II Turbo 2.0 analyzer. Those samples were also analyzed by capillary electrophoresis (Capillarys 2; Sebia, Lisses, France) to confirm the absence of Hb variants. HbA_1c_ in the 40 samples without HA variants (4.4–14.4% HbA_1c_) were then analyzed with different assays. Furthermore, five blood samples without hemoglobin A expression obtained from previously tested clinical specimens underwent hemoglobin analysis by Sebia capillarys 2 system and the type of hemoglobin was confirmed by genotyping analysis. Five samples with compound heterozygotes were collected: the genotype of the first sample was αα/αα and β^CD26^/β^CD41-42^; the second sample was αα/αα and β^IVS2-654^/β^NewYork^; the third sample was similar to the second sample, αα/αα and β^CD41-42^/β^NewYork^; the fourth sample was αα/αα and β^CD41-42^/β^J-Bangkok^; the fifth sample was -SEA/-α^4.2-Q-Thailand^ and β/β. The above five patients were nondiabetic patients with normal fasting blood glucose. All blood samples were aliquot quadruplicate and frozen at −70°C before analysis.

### Analysis methods

In present study, Bio–Rad Variant II system (Bio–Rad, U.S.A) was used as a comparative method for Hb_1c_ measurement given, it gained National Glycohemoglobin Standardization Program Level Laboratory certification and was traceable to the Diabetes Control and Complications Trial Reference method. A total number of 40 normal samples (no Hb variants) and five samples from patients with compound heterozygotes were examined using five routine methods: (1) the HPLC Variant II system (Bio–Rad, U.S.A); (2) the HPLC Variant II Turbo 2.0 system (Bio–Rad, U.S.A); (3) the Ultra2 system (Trinity Biotech, U.S.A) using the BAC principle; (4) the Capillarys 2 Flex Piercing (C2FP) system (Sebia, France) using the CE principle; (5) the Roche Modular PPI system (Roche, Germany) using Tina-quant Hemoglobin A_1c_ III principle. The mixed whole blood samples were adopted to transferring value-assignment in order to improve the comparability of those systems to Bio–Rad Variant II. To analyze the results detected from samples with five examining assays, Bland–Altman plots were performed. Ordinary linear regression was performed to regression and bias analysis. The percentage deviation plots were used to carry out bias estimation (−6.0–6.0%).

### Statistical analysis

Statistical analyses were carried out using SPSS software version 17.0. The Student’s *t*-test was used to compare differences between two groups. Data were presented as means ± standard deviations (SDs). Statistically significant difference was defined as *P* value <0.05.

## Results

### HbA1c measurement in the patient without HA variants

**Figure 1:**
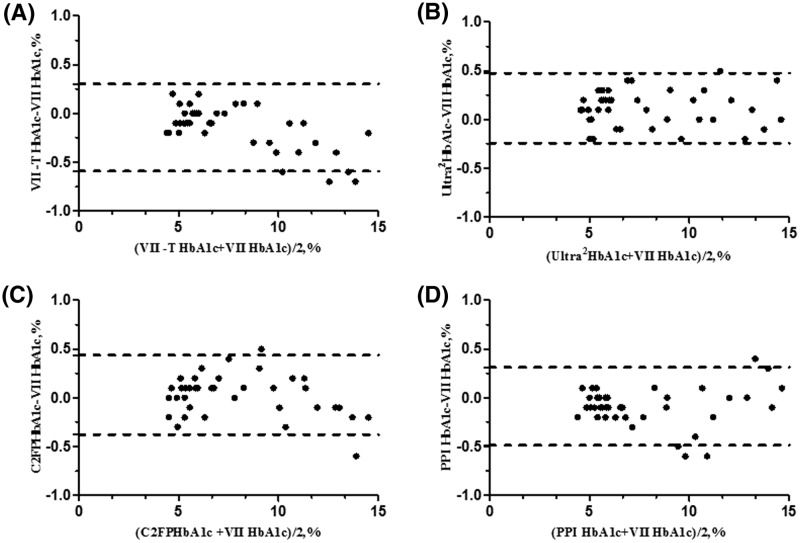
Bland–Altman difference plot applied to comparing HbA1c results of VII system with other systems *X*-axis is mean between the test and comparative methods (%). *Y*-axis is difference between the test and comparative methods (%). Dotted lines represent 95% Cl (**A**) VII-T system compares with VII system; (**B**) Ultra2 system compares with VII system; (**C**) C2FP system compares with VII system; (**D**) PPI system compares with VII system.

The HbA_1c_ values of 40 patients without HA variants were correlated with VII system detected by VII, VII-T 2.0, Ultra2, C2FP, and PPI system. The results were further analyzed with corresponding equations: *y* = 1.0514*x* − 0.2502 (*R*^2^ = 0.9969), *y* = 0.9991*x* − 0.1105 (*R*^2^ = 0.9965), *y* = 1.0178*x* − 0.1701 (*R*^2^ = 0.9957), and *y* = 0.9932*x* + 0.1404 (*R*^2^ = 0.9957). Bland–Altman plots (Figure 1) showed agreement between VII system and VII-T, Ultra2, C2FP, PPI systems and the 95% confidence interval (95% Cl) for the deviation between VII system and those systems was −7.5% to +7.5% (95% Cl VII-T: 0.3 to −0.59; Ultra2: 0.48 to −0.24; C2FP: 0.38 to −0.44; PPI: 0.49 to −0.31).

### HbA_1c_ measurement in the patients with compound heterozygotes

HbA1c is formed on the basis of HbA and the change of HbA can impact the formation of HbA1c. Blood samples without HbA are defined as no HbA1c expression according to the definition of the International Federation of Clinical Chemistry and Laboratory Medicine. Five blood samples with compound heterozygotes in our study were detected without HbA expression, indicating that there was no HbA1c expression. However, the five examining systems showed different results.

**Figure 2:**
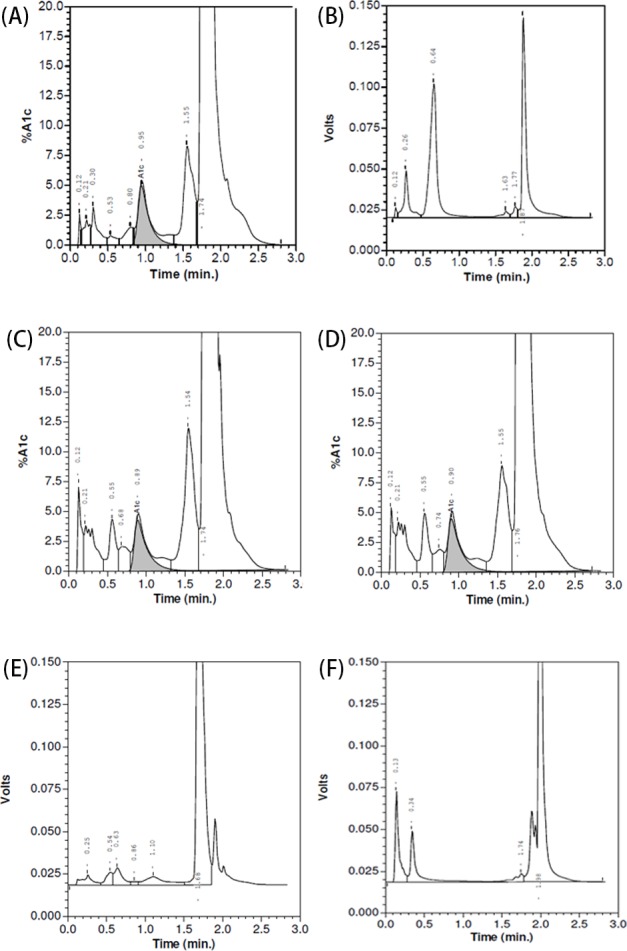
The chromatogram of five double heterozygous carriers on Bio–Rad VⅡ analyzer (**A**) Normal sample; (**B**) β^CD26^/β^CD41-42^carrier; (**C**) β^IVS2-654^/β^NewYork^ carrier; (**D**) β^CD41-42^/β^NewYork^ carrier; (**E**) β^CD41-42^/β^J-Bangkok^ carrier; (**F**) –SEA/-α^4.2-Q-Thailand^ carrier.

**Figure 3:**
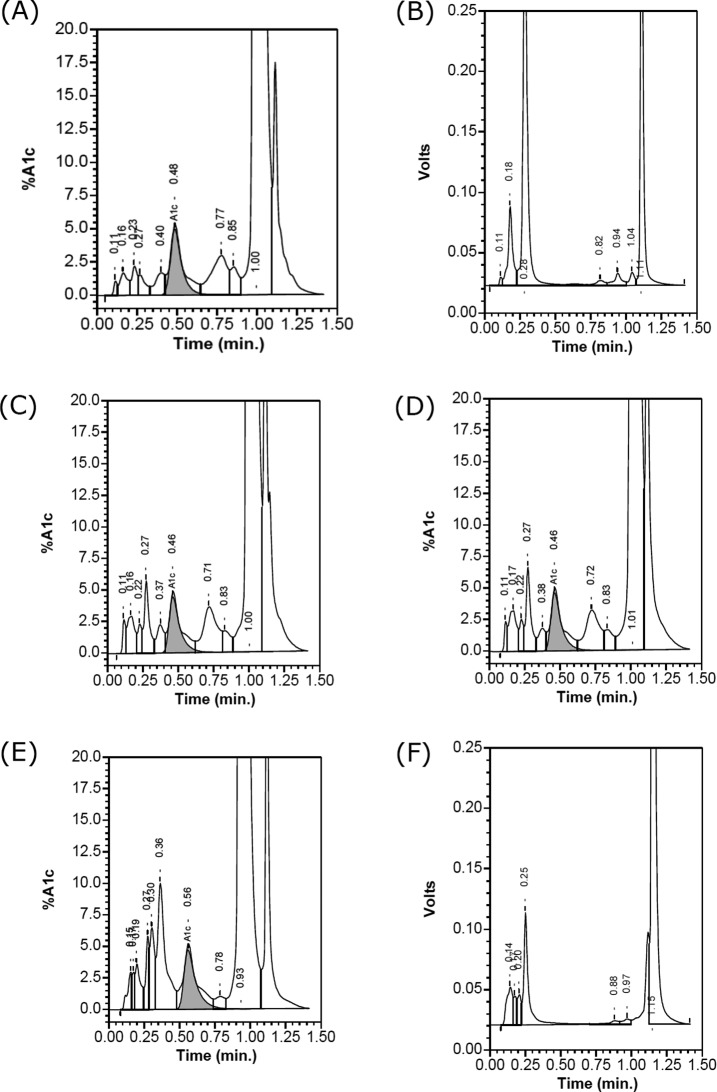
The chromatogram of five double heterozygous carriers on Bio–Rad VⅡ Turbo analyzer (**A**) Normal sample; (**B**) β^CD26^/β^CD41-42^carrier; (**C**) β^IVS2-654^/β^NewYork^ carrier; (**D**) β^CD41-42^/β^NewYork^ carrier; (**E**) β^CD41-42^/β^J-Bangkok^ carrier; (**F**) -SEA/-α^4.2-Q-Thailand^ carrier.

Two erroneous results for HbA1c were attained in the VII analyzer and the chromatograms of the first (Figure 2B), fourth (Figure 2E), and fifth (Figure 2F) samples were all labeled ‘Volts’, which meant abnormal electrophoresis signal and was considered unacceptable according to the manufactures’ instructions. However, the VII chromatograms for the second (Figure 2C) and third (Figure 3D) samples with Hb New York and β-thalassemia (Figure 2C,D) were not easily distinguished from a normal HbAA chromatogram (Figure 3A) and the proportions of the HbA1c (Table 1) and HbA0 (85.5% and 86.3% respectively) were produced. The peak of Hb NewYork0 was all mistakenly identified as HbA0.

**Table 1:** HbA1c values for five double heterozygotes carriers on Bio–Rad VII, Bio–Rad VII-T 2.0, Sebia Capillarys 2 Flex Piercing,Trinity Biotech Ultra2, and Roche PPI systems

Samples	Genotypes	Bio–Rad VII	Bio–Rad VII-T	Sebia C2FP	Trinity Biotech Ultra2	Roche PPI
1	αα/αα; β^CD26^/β^CD41-42^	Nr	Nr	Nr	4.2%	4.5%
					22 mmol/mol	26 mmol/mol
2	αα/αα; β^IVS2-654^/β^NewYork^	4.3%	4.5%	Nr	4.1%	4.2%
		23 mmol/mol	26 mmol/mol		21 mmol/mol	22 mmol/mol
3	αα/αα; β^CD41-42^/β^NewYork^	4.5%	4.6%	Nr	4.3%	4.8%
		26 mmol/mol	27 mmol/mol		23 mmol/mol	29 mmol/mol
4	αα/αα; β^CD41-42^/β^J-Bangkok^	Nr	4.7%	Nr	4.7%	3.8%
			28 mmol/mol		28 mmol/mol	–
5	-SEA/-α^4.2-Q-Thailand;^ β/β	Nr	Nr	3.9%	5.3%	5.7%
				–	34 mmol/mol	39 mmol/mol

Nr, no HbA1c value was reported for this sample by the system; ‘–’, no IFCC HbA1c value transferred.

Similarly, the chromatograms of the VII-T 2.0 analyzer for the first (Figure 3B) and fifth (Figure 3F) samples also did not show the results of HbA1c and also labeled ‘Volts’. The VII-T 2.0 chromatograms for the second (Figure 3C) and third samples (Figure 3D) also appeared normal with no indication of variants and the Hb New York appeared to elute as the HbA0 window. The VII-T 2.0 chromatogram for the fourth (Figure 3E) was different from the VII chromatogram and showed a HbA1c value (4.7% in Table 1). Checking the chromatogram, we found the proportion of P4 was very high (84.9%), which was different from normal chromatogram (under 1.5%). So, we assumed that the P4 window was the peak of Hb J-Bangkok.

**Figure 4:**
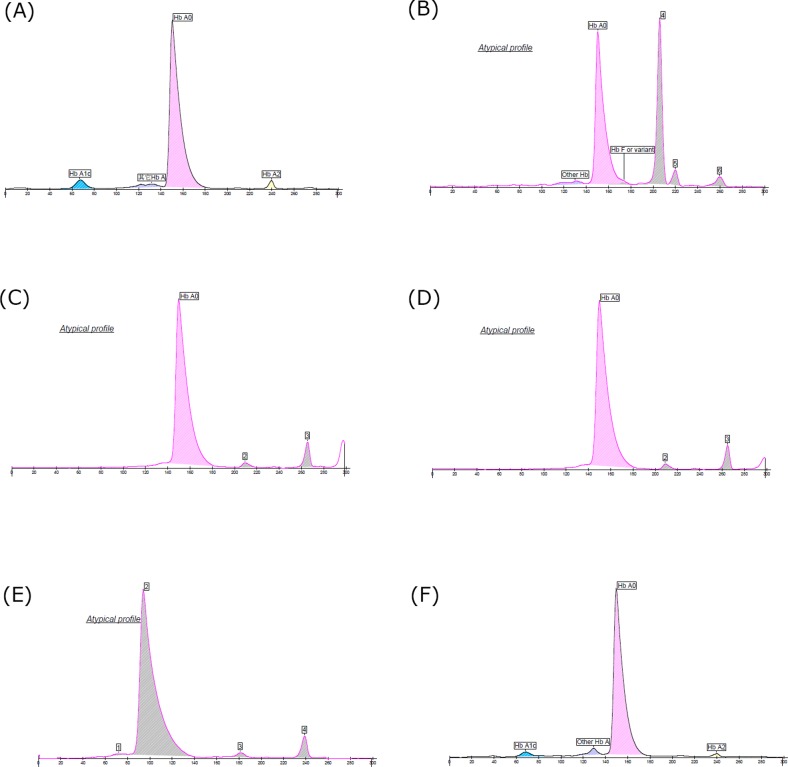
The chromatogram of five double heterozygous carriers on Sebia C2FP analyzer (**A**) Normal sample; (**B**) β^CD26^/β^CD41-42^carrier; (**C**) β^IVS2-654^/β^NewYork^ carrier; (**D**) β^CD41-42^/β^NewYork^ carrier; (**E**) β^CD41-42^/β^J-Bangkok^ carrier; (**F**) -SEA/-α^4.2-Q-Thailand^ carrier.

Of the five heterozygotes analyzed by C2FP system, erroneous results were reported in only one sample (Figure 4F). The electropherograms for the first, second, third and fourth, (Figure 4B–E) all showed the ‘atypical profile’ flag due to identification of the abnormal electropherograms. In addition, four of five electropherograms misidentified the peak of Hb variants as the peak of Hb A0. For the fifth sample with Hb Q-H disease (Figure 4F), electropherogram of C2FP analyzer produced an erroneous ‘normal’ HbA1c (3.9% in Table 1) result and did not show the peak of HbH.

All of those hemoglobin variants were undetectable on the HbA1c principle from PPI and primus Ultra2 systems. It was remarkable that Ultra2 and PPI systems all produced spuriously ‘normal’ HbA1c results for all five double heterozygotes (Table 1).

## Discussion

The HbA_1c_ value reflects the patient’s mean glycemic level in the past 6–8 weeks. Hemoglobinopathy alters the composition and structure of hemoglobin and may lead to misinterpretation of the HbA_1c_ result. Hemoglobin variants have been reported to potentially affect the precision of current examining methods for HbA_1c_ measurement [10–12]. However, HbA_1c_ values measured in patients with compound heterozygotes are rarely reported.

HbA_1c_ has been referred as one of major markers for diabetes diagnosis by the World Health Organization since 2011 [13]. However, HbA_1c_ is still not a diagnostic criterion for diabetes in China yet. One of the reasons is that China is a population with a high prevalence of hemoglobinopathy and thalassemias [14–17]. The incidence of α- and β-thalassemias is 8.53% and 2.54% respectively [14]. Of note, the incidence of abnormal Hbs is 0.358% in Chaozhou city [15] and 0.4% in Dongguan city, China. The common α-globin variants are Hb Constant Spring, Hb Q-Thailand, and Hb G-Honolulu. The common β-globin variant was Hb E, Hb New York, Hb J-Bangkok, et al. [16]. Thalassemias combined with hemoglobin variants are a special physiopathological condition that cannot form HbA. Five cases in our article were double heterozygous samples that do not contain HbA and HbA_1c_. We tested those five samples by five examining systems in order to assess the specificity of common methods in the measurement of HbA_1c_.

HbA_1c_ values can be evaluated by various methods based on the molecular charge (cation-exchange high-performance liquid chromatogram (CE-HPLC) and electrophoresis) or molecular structure (immunoassays, boronate affinity chromatography, and mass spectrometry). Both VII and VIIT2.0 belong to CE-HPLC assay, which can separate HbA_1c_ from HbA because glycation of the N-terminal valine reduces the positive charge. In present five cases, the two principles reported two of five and three of five spurious HbA_1c_ results respectively. Because Hb New York has the similar charge to HbA and can be eluted together with HbA Hb New York in the second and third cases, it was erroneously identified as HbA0 due to elution in the respective retention and Hb (New York)_1c_ misidentified as HbA_1c_. Hb J-Bangkok is another common hemoglobin variant in China and its charge is different from HbA. Therefore, the chromatograms of VII and VIIT2.0 were shown abnormally. Lo et al. [18] reported that a case of spuriously normal HbA_1c_ results due to misidentifying HbG Taipei as HbA0 by the variant II system. These results showed that CE-HPLC was obviously interfered by Hb variants with different charges and might result in erroneous HbA_1c_ concentration.

C2FP is a new assay to evaluate HbA_1c_ based on the separation of Hb property and charge by capillary electrophoresis. There is a strong consistency between the results of C2FP and VII [19]. Many literatures have reported that the resolution of C2FP is superior to CE-HPLC resulting from allowing the separation of many common and rare Hb variants from the HbA0 fraction [19–21]. Of five samples without HbA expression, C2FP system could detect HbA_1c_ up to four samples. Although C2FP system misidentified Hb F as HbA0 in the first sample and Hb (New York) 0 as HbA0 in the second and third sample, the system did not display HbA_1c_ values, which might prompt laboratories to pay more attention to those patients with hemoglobin fractions. However, in the fifth sample, C2FP system detected the HbA_1c_ values without showing HbH expression and misidentified HbQ-Thailand as HbA0. Literatures had reported that C2FP system produced two inaccurate results of the 18 rare variants (Hb Silver Springs and Hb J-Broussais) [11]. For HbG Coushatta, C2FP system produced a HbA_1c_ value different from the results detected by Tandem HPLC-capillary electrophoresis (IFCC reference method) [22]. Although C2FP system could separate many common and rare Hb variants from the HbA0 fraction, we also must focus on analyzing raw data of the different Hb variants and finding the problems of the electropherograms.

Ultra2 system using boronate affinity HPLC method is based on the fact that glycated and non-GHB are separated regardless of hemoglobin species and the Ultra2 system for Hb A_1c_ seems to have less interference by Hb variants compared with the IFCC Reference Method [23]. PPI system is based on Tinaquant immunoassay using antibodies to target N-terminal glycated amino acids on the β chain for quantifying Hb A_1c_ and the Hb A_1c_ percentage is calculated according to the Hb A_1c_ and Hb concentrations. Ultra2 system and Tinaquant immunoassay had been used as comparative methods in previous articles since these methods were less likely to show interference by Hb variants [11,24]. In our five cases, the Ultra2 system and PPI system measured total glycated hemoglobin and detected a normal HbA_1c_. But there was no Hb A in these samples and Hb A_1c_ could not be detected. So, we assumed that the HbA_1c_ measurement derived from patients without Hb A expression by those examining systems should not be used as an indicator of those people with average blood glucose level to screen and diagnose the diabetes mellitus.

## Conclusions

Each examining system for HbA_1c_ measurement could not eliminate the interference of double heterozygous in our work. The implementation of such methods may not generate trustworthy results for clinic application. Though C2FP system may be superior to other systems, it is important to know that the hemoglobinopathy can affect HbA_1c_ measurement. Patients with compound heterozygous variants are suggested to use non-Hb-based method, such as fructosamine, glycated albumin or continuous glucose monitoring, to assess long-term glycemic control instead of Hb A_1c_ measurement. Due to the high frequencies of hemoglobin variants, it is important to clarify these limitations when using these methods to measure Hb A_1c_.

## Abbreviations

BAC: boronate affinity high-performance liquid chromatography
C2FP: Capillarys 2 Flex Piercing
CE: capillary electrophoresis
CE-HPLC: cation exchange-high performance liquid chromatography
HbA_1c_: glycated hemoglobin
